# Impact of Socioeconomic Factors and Lifestyle on Salt and Potassium Intake and Sodium-to-Potassium Ratio: EH-UH 2 Study

**DOI:** 10.3390/nu18040615

**Published:** 2026-02-13

**Authors:** Mihaela Marinović Glavić, Matea Bilobrk, Lovorka Bilajac, Andrej Belančić, Marta Bolješić Dumančić, Marija Domislović, Mirjana Fuček, Ana Jelaković, Josipa Josipović, Jagoda Nikić, Ivan Pećin, Ana Stupin, Petar Šušnjara, Željko Reiner, Bojan Jelaković

**Affiliations:** 1 Department of Social Medicine and Epidemiology, University of Rijeka, Faculty of Medicine, 51000 Rijeka, Croatia; lobilajac@gmail.com (L.B.); ana.jelakovic9@gmail.com (A.J.); 2 Croatian Hypertension League, 10000 Zagreb, Croatia; mateabilobrk5@gmail.com (M.B.); a.belancic93@gmail.com (A.B.); boljesic1997@gmail.com (M.B.D.); domislovic.marija@gmail.com (M.D.); mirjana.fucek@kbc-zagreb.hr (M.F.); josipa.josipovic01@gmail.com (J.J.); jagodanikic@yahoo.com (J.N.); ivanpecin@yahoo.com (I.P.); ana.stupin@mefos.hr (A.S.); psusnjara@kifos.hr (P.Š.); zeljko.reiner@kbc-zagreb.hr (Ž.R.); 3 Teaching Institute of Public Health Primorje—Gorski Kotar County, 51000 Rijeka, Croatia; 4 Department of Public Health, Faculty of Health Studies, University of Rijeka, 51000 Rijeka, Croatia; 5 Department of Basic and Clinical Pharmacology and Toxicology, University of Rijeka, Faculty of Medicine, 51000 Rijeka, Croatia; 6 Department of Anatomy and Neuroscience, Faculty of Medicine, University of Osijek, 31000 Osijek, Croatia; 7 Department for Nephrology, Hypertension, Dialysis and Transplantation, University Hospital Centre Zagreb, 10000 Zagreb, Croatia; 8 Department of Laboratory Diagnostics, University Hospital Centre Zagreb, 10000 Zagreb, Croatia; 9 Department of Nephrology and Dialysis, Sestre Milosrdnice University Hospital Centre, 10000 Zagreb, Croatia; 10 School of Medicine, Catholic University of Croatia, 10000 Zagreb, Croatia; 11 Nursing School Mlinarska, 10000 Zagreb, Croatia; 12 Department for Metabolic Diseases, University Hospital Centre Zagreb, 10000 Zagreb, Croatia; 13 School of Medicine, University of Zagreb, 10000 Zagreb, Croatia; 14 Department of Physiology and Immunology, Faculty of Medicine Osijek, University of Osijek, 31000 Osijek, Croatia; 15 Scientific Centre of Excellence for Personalized Health Care, University of Osijek, 31000 Osijek, Croatia; 16 Faculty of Kinesiology Osijek, Josip Juraj Strossmayer University of Osijek, 31000 Osijek, Croatia

**Keywords:** lifestyle, potassium, salt, socioeconomic factors, sodium-to-potassium ratio, 24-h urine sample

## Abstract

Background: There are conflicting reports on the association of socioeconomic (SES) characteristics and lifestyle with salt and potassium intake as well as with the sodium-to-potassium (Na/K) ratio. This paper examined how SES status and lifestyle habits affect salt, potassium intake, and the Na/K ratio in adults. Methods: Adults subjects (random sample) from the EH-UH 2 nationwide study with valid 24 h urine samples were included in these analyses. We used a questionnaire which included SES and questions related to lifestyle. Salt and potassium levels were measured using a 24 h urine collection. Results: A low level of professional qualification and education are important predictors of high salt and low potassium intake. SES affects salt intake more than potassium intake. Processed meat was the most important determinant of high salt intake. It significantly affected potassium intake, but this was not relevant due to a poor Na/K ratio. Non-smoking status was related to high daily salt and potassium intake, but with no significantly positive impact on Na/K ratio. Former smokers swapped one unhealthy habit for another, such as overeating or consuming too much salt. The Adriatic/Mediterranean diet, represented in this study with frequent olive oil and fish consumption, was related to more favourable salt and potassium intake and a better Na/K ratio. Targets of daily salt and potassium intake, as well as of Na/K ratio were achieved in a very low proportion of the population regardless of SES, lifestyle and behaviour. Conclusions: Our results emphasize the need for public-health strategies that consider both diet and individual characteristics to address nutritional inequalities and promote healthier eating habits. Targeted nutrition programmes for lower SES groups should emphasize salt reduction and encourage potassium-rich diets, thus reducing health imparities and the burden of diet-related chronic diseases. The prevention strategy should be more proactive and specifically designed for the food (meat) industry. A more holistic approach should be taken for smokers when quitting smoking is necessary, the whole population should be educated to change habits toward the Adriatic diet pattern, and the government should make olive oil and fish more affordable to all citizens, particularly to those with poor SES.

## 1. Introduction

### Salt and Potassium Consumption, Lifestyle Factors, and Socioeconomic Status

Excessive salt intake is strongly associated with high blood pressure (BP), cardiovascular (CV) diseases and premature deaths worldwide [1,2,3,4,5,6,7,8,9,10,11]. The World Health Organization (WHO) recommends that daily salt intake should be less than 5 g per day. While reducing salt intake is regarded as the most effective strategy for preventing CV disease and related deaths, salt consumption still exceeds recommended levels [9,10,11]. Globally, dietary potassium intake is well below the WHO recommendation of 3.5 g per day, with just 14% of people meeting this guideline [12,13]. Both salt reduction and potassium intake are recommended by relevant guidelines for hypertension [14,15]. It was shown that the sodium-to-potassium (Na/K) ratio is a better predictor of high BP and hypertension than either sodium or potassium alone, which further supports the idea of an overall dietary pattern that reduces the risk of hypertension [16,17,18,19].

Socioeconomic conditions substantially influence food choices and overall dietary composition, including sodium and potassium consumption. Research consistently indicates that people from lower socioeconomic backgrounds are more likely to follow dietary patterns marked by excessive sodium intake and insufficient potassium intake compared with more advantaged groups. These differences arise from inequalities in access to healthy foods, financial resources, and nutrition-related knowledge. Individuals with lower socioeconomic status often depend on low-cost, highly processed food products that contain large amounts of sodium and limited quantities of potassium, while opportunities to consume fresh fruits and vegetables are more restricted. The level of education represents a key socioeconomic factor shaping dietary behaviour. Higher educational attainment is commonly associated with a better understanding of dietary recommendations, greater engagement with food labelling, and healthier nutritional practices, such as moderating salt intake and increasing consumption of foods naturally rich in potassium. In contrast, lower educational levels are linked to a greater intake of convenience foods and industrially processed products that are typically sodium-dense and potassium-poor. These contrasting patterns contribute to enduring differences in cardiovascular risk between population groups [20,21,22,23,24]. Economic resources and employment conditions also play an important role in determining nutrient intake. Limited household income may constrain the ability to purchase fresh and nutritionally balanced foods, leading to greater reliance on cheaper alternatives that are often high in sodium. Moreover, demanding work schedules, shift work, or physically intensive occupations can encourage the consumption of fast foods and pre-prepared meals, further increasing sodium exposure and reducing potassium intake. Such circumstances contribute to an unfavourable sodium–potassium balance and help explain observed socioeconomic gradients in blood pressure and cardiovascular disease outcomes [25,26]. Broader social and neighbourhood conditions, beyond individual socioeconomic factors, also influence how much sodium and potassium people consume. Residents of socioeconomically disadvantaged areas often face limited access to fresh, healthy foods and greater exposure to fast-food restaurants and convenience stores, environments commonly described as “food deserts” or “food swamps”. These settings encourage eating patterns high in sodium and low in potassium, leading to an unhealthy sodium-to-potassium ratio. Research in large populations shows that neighbourhood deprivation predicts higher sodium intake and lower fruit and vegetable consumption independently of a person’s own income or education, highlighting the additional impact of contextual socioeconomic factors on dietary choices [23,27,28].

The ratio between sodium and potassium intake has been proposed as a more sensitive marker of cardiovascular risk than either nutrient considered independently. An elevated ratio reflects the combined effect of excessive sodium consumption and inadequate potassium intake, a pattern that is more frequently observed among socially and economically disadvantaged populations. Previous investigations have demonstrated that higher sodium-to-potassium ratios are associated with increased blood pressure and a greater likelihood of cardiovascular events. For this reason, exploring how socioeconomic factors impact both sodium and potassium intake, as well as their ratio, is critical for identifying dietary inequalities and for guiding effective public-health interventions aimed at reducing cardiovascular risk [29,30,31].

Several lifestyle habits, including physical activity, smoking, and dietary patterns, have been found to be associated with both salt and potassium intake [32,33,34]. Lifestyle habits often align with socioeconomic status, tailoring how much sodium and potassium people consume. Those with fewer socioeconomic resources tend to engage in several unhealthy behaviours (being physically inactive, smoking, drinking more alcohol) which are linked to a greater intake of processed, calorie-dense foods [35,36,37].

Physical inactivity, smoking and alcohol consumption often occur alongside diets low in fruits and vegetables, key sources of potassium, further tipping the nutrient balance toward too much sodium and too little potassium. Alcohol use and smoking, which are more common in lower socioeconomic groups, are also linked to poorer overall diet quality. This often means eating more processed foods high in sodium and fewer foods that naturally provide potassium [38]. In contrast, people who follow healthier routines, including regular exercise and dietary patterns like the Mediterranean or DASH diet, typically consume less sodium, more potassium, and achieve a more favourable sodium-to-potassium ratio. These clusters of behaviours accumulate to influence cardiovascular risk and help clarify why imbalances in sodium and potassium intake are more common in socially and economically disadvantaged groups [39,40,41].

Salt consumption decreased significantly in Croatia over the past decade [42,43]. However, in a majority of the population, salt ingestion was above, and potassium intake below, recommended values. Consecutively, the Na/K ratio was far from the recommended value of <1. We also reported on better salt and potassium intake in the Mediterranean part of Croatia compared to the continenatal regions, reflecting some cultural, social and culinary differences among regions [42]. The aim of this paper was to further analyze the impact of SES and lifestyle beahviours on salt and potassium intake and the Na/K ratio, and with achieved recommended targets.

## 2. Participants and Methods

### 2.1. Study Design

This cross-sectional study was conducted as part of the EH-UH 2 study which investigates the epidemiology of hypertension and salt intake in Croatia and constitutes a nationally representative sample of non-institutionalized adults. The study involved comprehensive collection of anthropometric, demographic, lifestyle, and biological marker data to assess risk factors related to cardio–kidney–metabolic health and to determine the prevalence of major chronic non-communicable diseases. A total of 2021 subjects were randomly selected from the general population using randomisation codes derived from family physicians’ patient registries. These codes corresponded to the unique identification numbers assigned to each individual, facilitating random selection and enabling planned follow-up. Examinations were scheduled on Fridays and Saturdays, with single 24 h urine collections obtained on regular weekdays (Thursday or Friday). The research protocol comprised three main phases: (a) administration of structured questionnaires, (b) physical measurements, and (c) collection of blood samples, spot urine, and 24 h urine specimens. All personnel involved in data collection, the mobile examination team, including nurses, physicians, pharmacists, fellows, residents, and medical students, were trained to perform study procedures uniformly and in accordance with standardized methods. As the results presented in this paper are part of a large national study, the methodology, which is identical to that used here, has been explained in detail in previously published papers [42,44].

### 2.2. Questionnaire

Subjects completed a questionnaire that included sociodemographic data and questions related to lifestyle, such as smoking, alcohol consumption, frequency of physical activity, and frequency of consumption of processed meat products, red meat, poultry, fish, and olive oil, and the regularity of physical activity. Socioeconomic factors assessed in the study included monthly family income, professional qualifications, and educational attainment level. Questions were answered using a Likert scale: not consuming at all, very rarely, twice per week, or consuming every day/almost every day. The scale for the frequency of consumption of olive oil and lard were as follows: 1 (first choice)–4 (fourth choice). After completing the questionnaire, the subjects participated in outpatient clinical examinations, and they where were required to bring a 24 h urine sample. All laboratory analyses were performed at the Central Laboratory in University Hospital Centre Zagreb (Ulica Mije Kišpatića 12, 10000, Zagreb, Croatia).

### 2.3. 24 h Urine Sample

Subjects received 2.5-litre polyethylene plastic containers and were given clear verbal instructions and comprehensive written directions for collecting a 24 h urine sample, emphasizing the importance of collecting every drop. They were instructed to discard the first urine of the day at the start of the collection, then collect all urine for the next 24 h, finishing with the first urine void the following morning. Subjects received a reminder call the day before the outpatient visit about the correct collection procedure. At the outpatient appointment, the team measured and recorded the total urine volume, mixed the sample thoroughly, and divided it into three 2 mL aliquots, which were kept at 4 °C and sent out the same day.

### 2.4. Ethical Considerations

Before participating in this study, each subject received information about the study and voluntarily signed a consent form. The survey is carried out in accordance with the Declaration of Helsinki and Good Clinical Practice [45].

### 2.5. Statistical Analysis

All collected data were entered into Microsoft Excel and subsequently analyzed using the IBM SPSS Statistics version 28.0.0.0 (IBM Corporation, Armonk, NY, USA) and R version 4.5.2 (R Foundation for Statistical Computing, Vienna, Austria). The normality of data distribution was tested with Kolmogorov–Smirnov test. The Kruskal–Wallis test was used for three or more group analysis. The results were reported as mean (standard deviation—SD), median (interquartile range—IQR), or percentages, as appropriate. Spearman’s rank correlation coefficient was applied to assess the relationship between variables (Supplementary Materials Figures S1 and S3). In addition to correlation analysis, linear regression models were performed for daily salt and potassium intake. Furthermore, multinomial logistic regression analyses were conducted to identify the most significant predictors of increased salt intake and reduced potassium intake.

To visually summarize the correlation structure and highlight patterns within the dataset, heat maps were generated, providing an intuitive representation of the magnitude and distribution of associations across all measured parameters (Supplementary Materials Figures S2 and S4). More intensive colours indicate higher prevalence, and less intensive colours indicate lower prevalence of subjects in specific a subgroup. In the heatmap, the figure includes three categories of daily salt intake (<5, 5–10, >10 g/day) and daily potassium intake (<3, 3–3.5, >3.5 g/day), defined in relation to the World Health Organization’s recommendations. The third block displays three sodium-to-potassium ratio categories: <1, 1–2.5, and >2.5. Several sensitivity analyses were conducted. A *p*-value < 0.05 was considered significant for all the results.

## 3. Results

### 3.1. Subject’s Characteristics

During the period from March 2018 to September 2021, a total of 2021 individuals were invited to participate, of whom 256 did not meet the inclusion or exclusion criteria. The remaining 1765 subjects were enrolled after randomization, provided informed consent, and participated in the home visit. Among them, 423 did not attend the outpatient visit, leaving 1342 subjects who attended the outpatient visit. Of these, 74 did not collect a 24 h urine sample. Consequently, 1268 subjects provided 24 h urine collections. After quality control, 201 samples were excluded due to insufficient urine volume (<500 mL; *n* = 104) or urine creatinine values outside the predefined acceptable range (*n* = 97). The final analytical sample therefore consisted of 1067 subjects, with men making up 35%. All subjects were 18+ with an age range of up to 89 and signed informed consent prior to participating in the study [42].

### 3.2. Salt and Potassium Intake, Sodium-to-Potassium Ratio and Socioeconomic Factors

Table 1 provides data regarding salt and potassium intake, as well as the Na/K ratio, among subjects categorized by average monthly family income, professional qualifications, and educational attainment. Subjects in two groups with the highest monthly family income had significantly higher salt intake than subjects in groups with lower income (*p* = 0.004). They also had significantly better Na/K ratios (*p* < 0.001). Subjects with more than 12 years of education had significantly better Na/K ratios than less-educated subjects (*p* < 0.001). The most educated group had the significantly lowest salt and the highest potassium intake compared to all other subgroups (*p* < 0.001). The low-skilled workers had the highest salt intake and worst Na/K ratio (*p* < 0.001 for both). There were no differences in potassium intake among groups of subjects analyzed by family monthly income, education level or professional qualification (*p* > 0.05). Overall, the low correlation and determination coefficients (Figure S1) indicate that, while the direction of associations is consistent with dietary expectations, the magnitude of these relationships is modest, and the dietary scores alone are limited predictors of sodium excretion and Na/K ratio at the individual level. Figure S2 presents a heat map of subject distribution (numbers and percentages) by daily salt intake, potassium intake, and Na/K ratio across sociodemographic categories. Positive trends appeared across all SES factors, with the strongest increase seen in the average family monthly income, and the weakest in education level. Subjects with lower income, less education, and lower professional qualifications were more likely to have high salt intake (≥10 g/day), low potassium intake (≤3.5 g/day), and an unfavourable Na/K ratio (≥2.5). Conversely, subjects with higher income, higher professional qualifications and longer education tended to more frequently have the recommended intake of salt and potassium as well as Na/K ratio. However, across all SES factors, the proportion of subjects achieving the recommended Na/K ratio below 1 remains consistently very low. Potassium intake was least connected to the sociodemographic characteristics.

### 3.3. Salt and Potassium Intake, Sodium-to-Potassium Ratio and Lifestyle Habits

Table 2 shows salt and potassium intake and Na/K ratios for groups classified by smoking, alcohol use, and physical activity. Former smokers consumed more salt (*p* = 0.041) and potassium (*p* = 0.004) than never-smokers and current smokers. There were no differences in the Na/K ratio. There were no significant differences in salt and potassium intake among alcohol subgroups as well as there were no differences among physical activity subgroups. Subjects who reported very active physical activity had the highest potassium and lowest salt intake, but differences compared to other subgroups were not statistically significant. Subjects who reported being physically active more than twice per week had the highest potassium intake and a better Na/K ratio than those who were never or only occasionally active. Table 3 presents the results for salt and potassium intake, as well as the Na/K ratio, in subjects grouped according to their consumption of processed meat, red meat, poultry, fish, and olive oil. Subjects who consumed processed meat most frequently had the highest salt (*p* < 0.001) and potassium (*p* = 0.092) intake, but also the worst Na/K ratio (*p* = 0.004). Salt consumption decreased (*p* < 0.001), and potassium intake increased (*p* = 0.768) as fish consumption increased. The Na/K ratio decreased as fish consumption increased (*p* < 0.001). A similar trend was noted in olive oil consumption. More frequent olive oil consumption was related to the lower salt (*p* < 0.001) and higher potassium ingestion (*p* = 0.290), and better Na/K ratio (*p* < 0.001). Figure S3 shows correlations and Figure S4 heat map displays the number and percentage of subjects per category for daily salt intake, potassium intake, and Na/K ratio, based on dietary habits. Subjects who frequently consumed processed meat had higher sodium intake, lower potassium intake, and an unfavourable Na/K ratio. Subjects who rarely or never ate processed meat generally had lower sodium and higher potassium intake, and a better Na/K ratio. Regular olive oil users are more likely to meet the recommended Na/K ratios. Subjects with regular fish consumption typically exhibit reduced sodium intake, increased potassium intake, and a lower Na/K ratio.

Figure S1 presents key Spearman’s correlation coefficients between SES factors, dietary habits, salt and potassium intake, and the Na/K ratio. There was a strong positive correlation between olive oil consumption and the Na/K ratio (R = 0.34). In contrast, lard consumption showed a negative correlation with the Na/K ratio (R = −0.24). Table S1 summarizes the association and trends between salt and potassium intake and Na/K intake and SES characteristics and lifestyle habits. Higher levels of all SES characteristics were associated with lower salt intake and a better Na/K ratio. SES characteristics had the least impact on potassium intake. Olive oil and fish consumption were associated with positive trends of salt and potassium intake and Na/K ratio. Less consumption of processed meat was associated with lower salt intake and a better Na/K ratio, but not with higher potassium intake. Former smokers ingested more salt and had a worse Na/K ratio, but higher potassium intake. In contrast, current smoking status was associated with less salt and less potassium intake. More physical activity was related to higher potassium intake and better Na/K ratio and modest association with salt intake.

### 3.4. Determinants of Salt and Potassium Consumption and Na/K Ratio

As shown in Table 4, the final model in stepwise linear backward regression which including all SES and lifestyle variables (R^2^ = 0.106) for daily salt intake predictors such as education, professional qualification, smoking status, and consumption of processed meat, olive oil, and salad. An increase in one unit (one category) of education and qualification leads to a decrease in salt intake by 0.55 g/day and 0.56 g/day, respectively. An increase in one category of processed meat consumption and decrease in one category of olive oil consumption leads to an increase in daily salt intake by 0.82 g/day and 0.62 g/day, respectively. Smoking status decreased daily salt consumption by 0.48 g/day. The final linear regression model for daily potassium intake (R^2^ = 0.041) (Table 5) included predictors such as education, professional qualification, smoking, and consumption of processed meat, olive oil, and salad.

Table 6 displays a model related to recommended salt intake (≤5 g per day) (R^2^ = 0.127), incorporating all SES and lifestyle variables. Compared to the recommended salt intake, higher salt consumption was significantly associated with more frequent intake of processed meats (OR = 1.9), reduced consumption of olive oil (OR = 1.5), non-smoking status (OR = 1.4), and lower levels of educational attainment (OR = 1.3). Table 7 displays a model related to recommended potassium intake (greater than 3.5 g per day) (R^2^ = 0.041), incorporating all SES and lifestyle variables. Recommended potassium intake was linked to more frequent fish (OR = 1.5) and olive oil (OR = 1.3) consumption.

### 3.5. Differences in Proportion of Subjects with Achieved Targets Depending on Socioeconomic Status and Lifestyle Factors

**Figure 1:**
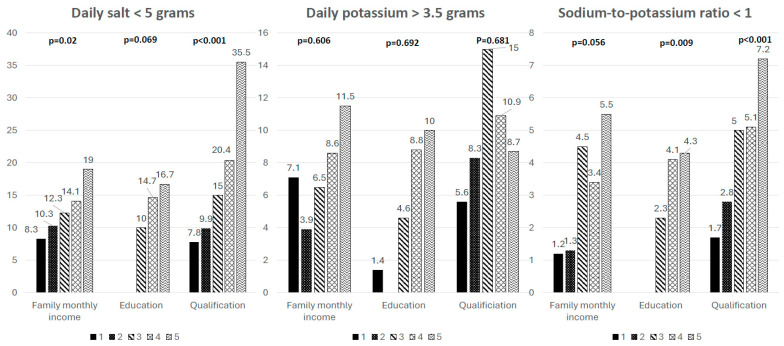
Differences in achieved targets of salt and potassium daily intake, and Na/K ratio in various SES groups. Family income: 1 < 2500 HRK, 2 = 2500–3500 HRK, 3–3500–5000 HRK, 4 = 5000–10,000 HRK, 5 > 10,000 HRK; Education: 1 = without formal education, 2 = <4 years, 3 = 4–8 years, 4 = 8–12 years, 5 = >12 years; Qualification: 1 = low-skilled worker, 2 = high-skilled worker, 3 = medium level of professional qualification, 4 = higher professional qualification, 5 = high professional qualification.

Figure 1 presents differences in proportions of subjects with achieved targets for daily salt and potassium intake and Na/K ratio related to the SES factors. The proportion of subjects who achieved any of targets is, in general, very low. Daily salt targets were achieved significantly more frequently than daily potassium targets in all SES categories.

**Figure 2:**
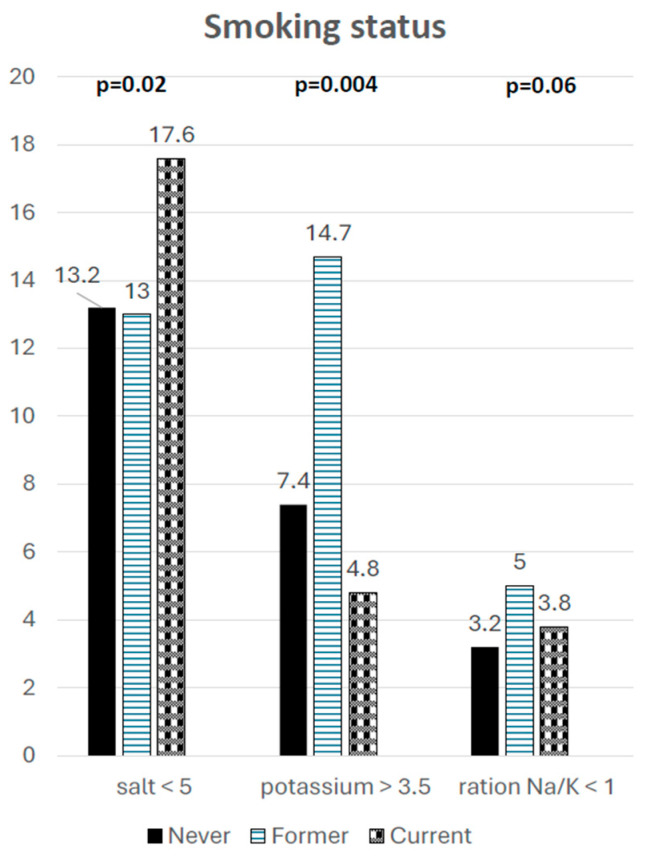
Differences in achieved targets of salt and potassium daily intake, and Na/K ratio regarding smoking status.

Compared to current smokers, former smokers are substantially less likely to achieve recommended salt intake levels. However, they are more likely to meet potassium intake targets, resulting in an improved Na/K ratio, which may indicate increased food consumption following smoking cessation (see Figure 2).

**Figure 3:**
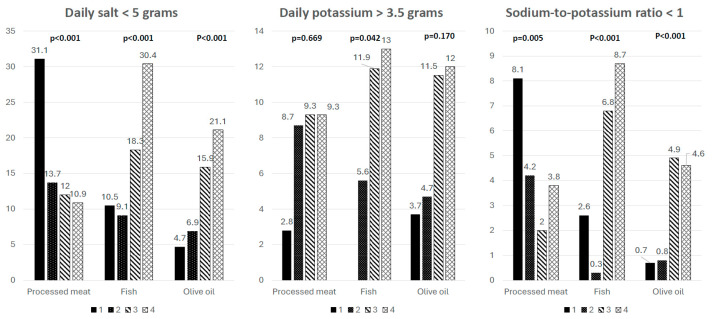
Differences in achieved targets of salt and potassium daily intake, and Na/K ratio regarding consumption of processed meat, fish and olive oil. 1 = never, 2 = very rarely (few times/month), 3 = up to twice/week, 4 = every day/almost every day; olive oil: 1 = first choice, 2 = second choice, 3 = third choice, 4 = fourth choice.

Achieving salt intake targets was associated with less frequent consumption of processed meat (Figure 3). Achieved potassium targets was found in subjects who more frequently consumed processed meat, but targets of the Na/K ratio were achieved more frequently by those who consumed processed meat less frequently. The frequency of fish and olive oil consumption was significantly more frequently associated with achieving target values for potassium intake, salt intake, and Na/K ratio. The frequency of salad, fruit, and vegetable consumption was not significantly associated with achieving target values (Figure 3). 

## 4. Discussion

This research supports earlier findings that SES factors and lifestyle habits are linked to salt, potassium intake, and the Na/K ratio. Our finding on the significant association of the higher frequency of oil and fish consumption with lower sodium and higher potassium consumption and a better Na/K ratio is the novelty, and we failed to find such information in other studies. An interesting finding, differing from some previous research, is that smokers consumed the least salt and potassium, while former smokers consumed the most.

### 4.1. Sociodemographic Characteristics

Higher average family monthly income and higher personal qualification were associated with lower salt intake and better Na/K ratio and vice versa. These patterns are consistent with the Italian MINISAL study, which reported that lower educational levels and manual occupations were associated with higher salt intake [46]. The Japanese study showed that lower household expenditure and shorter educational attainment were associated with lower potassium excretion and a higher Na/K ratio, while SES differences in sodium intake were less consistent [21]. Results from the other Japanese study showed that SES disparities were far more evident for potassium intake and the Na/K ratio than for sodium [28]. Cultural distinctions between Asia and Europe may account for the differences observed between Japanese studies and European research, including our own. A systematic review and meta-analysis further reinforce the clear SES gradient in salt intake. Across fifty-one studies from nineteen high-income countries, subjects with lower SES consistently consumed more salt than those with higher SES. The study found that SES differences in sodium intake likely worsen chronic disease rates among disadvantaged groups [47,48]. The financial constraints may limit the ability of lower-SES groups to meet potassium recommendations and achieve a favourable Na/K ratio [49]. Our findings are in concordance with this statement. Potassium intake was similar across income groups but slightly higher in those with the highest incomes, likely because they consumed more fruits, vegetables, and other potassium-rich foods. This could not reflect only higher purchasing power but also better health literacy. The observed better Na/K ratio in subjects with higher SES were related more to lower salt than to higher potassium intake. Although the patterns of salt and potassium intake and the Na/K ratio were the best in the highest SES categories, it is still far from the WHO’s recommendations. In summary, SES affects salt intake more than potassium intake, and an improved Na/K ratio results mainly from reduced salt consumption rather than increased potassium intake.

### 4.2. Lifestyle Habits

#### 4.2.1. Smoking Status

Smokers had a significantly higher Na/K ratio than non-smokers according to other authors, which is the opposite of our results [50]. Former smokers consumed more salt and potassium than current smokers, who had the lowest intake, likely because they ate less overall. We explained higher salt and potassium intake in former smokers with a higher general intake of food. Former smokers substituted and replaced one detrimental habit (smoking) by another (excessive food and salt intake). Smoking cessation should be holistic, educating former smokers about both its benefits and the need to manage other risk factors like obesity and salt intake.

#### 4.2.2. Alcohol Consumption

We failed to find significant differences in salt and potassium intake, as well as in sodium-to-potassium intake among subject groups based on the amount of alcohol consumption. The literature is scarce on this topic, but our results are in line with other authors who analyzed the general population with a 24 h urine collection [51,52].

#### 4.2.3. Physical Activity

In the present study, although physical activity was not significantly associated with sodium or potassium intake in the total sample, relevant patterns emerged: subjects who reported engaging in sport or regular exercise consumed less sodium, more potassium, and demonstrated the best Na/K ratio compared with those who were inactive or exercised only sporadically. Evidence from previous research illustrates substantial heterogeneity in this association across populations. Among Korean study participants, those in the highest tertile for Na/K ratio were more likely to report reduced levels of physical activity [53]. Kwon et al. found no evidence that sodium or potassium intake—or their ratio—interacted with physical activity to influence mortality risk, suggesting that exercise may exert benefits through mechanisms beyond electrolyte intake [54]. Opposing these findings, Park et al. showed that individuals in the highest sodium-intake quartiles were more likely to belong to the high physical activity group, suggesting a clustering of higher sodium consumption with more active lifestyles in certain populations [55].

#### 4.2.4. Processed Meat

A Chinese study found that processed foods contributed 38% of sodium intake, with many containing high sodium and a poor Na/K ratio [56]. In Northern Italy, processed meats accounted for about 25% of daily sodium intake, while potassium intake primarily came from fresh fruits and vegetables typical of the Mediterranean diet [57]. Processed foods not only contain high levels of sodium, but they also worsen the Na/K ratio because they rarely have enough potassium to offset the effects of sodium. This imbalance is a key factor in the risk for CV diseases associated with high sodium and low potassium intakes [58]. In the Irish study, processed meats, breads, and butter were consistently associated with a higher Na/K ratio, reflecting a dietary pattern richer in sodium relative to potassium. In contrast, greater consumption of fruits, vegetables and fresh meats was associated with lower Na/K ratio [59]. These findings agree well with ours. Salt and potassium intake were linked to how often processed meat was consumed, with the Na/K ratio rising as processed meat intake increased. The main contributor to a better Na/K ratio was associated with less consumption of processed meat and a lower salt intake, not a higher potassium intake.

#### 4.2.5. Fish and Olive Oil Consumption as an Adriatic Diet Paradigm

We found that salt consumption significantly decreased as fish consumption increased. The amount of potassium intake increased as the fish consumption increased, and finally the Na/K ratio decreased as fish consumption increased. A similar trend was seen with olive oil consumption. More frequent olive oil consumption was related to lower salt and higher potassium ingestion and a better Na/K ratio. While it might seem that increased potassium intake from eating more fish simply reflects the naturally high potassium content of fish, we suggest that eating more fish and olive oil points to an overall healthier diet—the Adriatic diet. This is in line with our previous report on significantly lower salt and higher potassium intake in the Mediterranean part of Croatia compared to the continental areas [42]. To the best of our knowledge, this is the first report on the association of fish and olive oil consumption with salt and potassium intake and the Na/K ratio.

### 4.3. SES and Lifestyle Predictors of Daily Salt and Potassium Intake

A very low R^2^ in stepwise backward regression indicates that our final models for both salt and potassium explain little variance, but significant predictors still show meaningful (though small) relationships. This is not strange for complex human behaviour studies like this one where context matters more than a single R^2^ threshold. Our model for salt confirmed that higher SES characteristics (education and professional qualification) were important predictors of both lower salt and higher potassium intake. We found that processed meat was the most important predictor of high salt intake which is in line with other authors [60,61]. Interestingly, we found that a higher intake of processed meat was also significant predictor of high potassium intake. Although meat and meat products, including processed meats, can contribute a portion of dietary potassium, they are not major sources of potassium compared with fruits, vegetables, legumes, and dairy, and diets high in processed meats are additionally characterized by a higher Na/K ratio, indicating poorer potassium status overall [62]. We found that smoking status was a predictor of both high salt and potassium intake. Former smokers tend to gain weight after quitting smoking due to increased food intake as nicotine’s appetite-suppressing effect disappears [63]. This can lead to higher potassium intake from fruits and vegetables, while sodium intake may remain high depending on food choices. As a result, former smokers are less likely to meet sodium targets but are more likely to achieve potassium goals, yielding a more favourable Na/K ratio than current smokers. The most intriguing result was that more frequent olive oil consumption was a significant predictor of low salt and high potassium intake. In line with our study results, in a Northern Italian population, higher adherence to a Mediterranean diet—characterized by a greater consumption of fish and olive oil—was associated with a significantly higher potassium intake and a lower Na/K ratio [57].

### 4.4. Achieved Targets SES and Lifestyle Predictors of Daily Salt and Potassium Intake

The proportion of subjects who achieved any of the targets in our cohort was, in general, very low. Daily salt targets were achieved significantly more frequently than daily potassium targets in all SES categories. Similar patterns have been observed in a population-level study in Oman based on 24 h urinary excretion where results showed that 22% of adults met the recommended salt intake, while fewer than 10% achieved the recommended potassium intake. This aligns with our findings and highlights the widespread difficulty in achieving adequate potassium intake [64]. In multinominal regression analyses, we found that achieving salt intake targets was associated with less frequent consumption of processed meat (Figure 3). Higher processed meat consumption is typically associated with higher sodium intake and worse adherence to recommended sodium targets in dietary studies [65]. In the study by Wu et al., higher processed meat consumption was initially linked with greater hypertension risk, but this association was substantially reduced and lost statistical significance after adjusting for sodium intake, suggesting that sodium content in processed meats largely explains the relationship [66]. Current smoking status was also predictor for achieving daily salt targets, but not of potassium targets, and this was already discussed. We found that more frequent olive oil consumption was a predictor of achieving both targets. Subjects who regularly use olive oil and fish tend to have healthier diets and a higher potassium intake. This was also shown by their Na/K ratio more often meeting recommended levels, which aligns with typical Mediterranean diets. Professional qualification was the key SES factor linked to meeting daily salt targets; no SES factors affected potassium intake in the final model.

### 4.5. Strengths and Limitations

This study has several important strengths. The study is based on data from a large, nationally representative sample of the adult Croatian population, which improves the generalizability of the findings. By using the gold standard method for assessing sodium and potassium intake, 24 h urine collection, measurement error was reduced compared to dietary questionnaires which are prone to recall bias, under-reporting, and imprecision in estimating [67,68]. Accordingly, reliance on 24 h urine collections in this study minimizes measurement error and provides a robust basis for estimating sodium and potassium intake at the population level. The research evaluated several SES indicators and various lifestyle factors to thoroughly examine how these elements, both independently and together, relate to salt intake, potassium consumption, and the Na/K ratio. The use of heat maps visually highlights the prevalence of undesirable intakes in low SES groups, improving interpretation for public health.

Our study has several limitations. The cross-sectional design prevents establishing causality, e.g., whether low SES causes poor intake or vice versa (reverse causation), and the lack of longitudinal data limits the assessment of changes over time. Questionnaires do not include detailed measures of fruit/vegetable intake (main sources of potassium). We used the Likert scale, as many other studies did. An SES index was not constructed for this analysis; rather, individual SES categories were examined separately. However, our aim was to analyze individual SES items. The 2018–2021 data collection excludes post-COVID dietary changes and focuses only on adults, omitting children and the elderly, where SES effects may be greater. The present analysis included a subgroup of 1067 subjects from the general EHUH 2 cohort. Although we made our results more robust and precise by including only those subjects with reliable (properly collected) 24 h urine samples, the question remained whether this may limit the generalizability of our findings. Accordingly, multiple sensitivity analyses were performed: outliers were excluded and only complete cases were analyzed with statistical methods tailored to the relevant distributional assumptions. We compared subjects with properly collected urine samples to those with unreliable samples. The observed differences highlight the importance of using only reliable samples, which strengthens our findings. Importantly, we analyzed differences between groups who completed the questionnaire, had laboratory data, and measurements with the group without all those parameters. We failed to find differences in gender, age and place of residency (continental vs. Mediterranean part of Croatia) which further supports the representativeness of our sample.

### 4.6. Implications of the Study

There are several possible implications of the study based on our results and conclusions: (i) subjects with poor SES had a higher risk for unfavourable salt and potassium intake—a prevention strategy should be tailored accordingly; (ii) processed meat was the most important determinant of high salt intake—a more active approach to the meat industry is needed; (iii) former smokers had a higher risk for high salt intake than current smokers. They swapped one unhealthy habit for another, such as overeating or consuming too much salt—a more holistic approach to smokers when quitting smoking is necessary; (iv) Adriatic/Mediterranean diet, represented in this study with frequent olive oil and fish consumption, was related to more favourable salt and potassium intake and a better Na/K ratio—the whole population should be educated to change habits toward this pattern, and the government should make these products more affordable to all citizens, particularly to those with poor SES. Our results emphasize the need for public-health strategies that consider both diet and individual characteristics to address nutritional inequalities and promote healthier eating habits.

## 5. Conclusions

Education and professional qualification, as important SES characteristics, and several lifestyle habits and behaviours were found to be strong predictors of low salt and high potassium intake. Across all SES and lifestyle groups, the proportion of individuals meeting recommended targets—especially for potassium intake and the Na/K ratio—was alarmingly low. Among included variables, processed meat was the strongest predictor of high salt intake and of achieved daily targets. The impact of processed meat on potassium intake was high but not relevant, as shown by the poor Na/K ratio. Non-smoking status was related to high daily salt and potassium intake, but with no significantly positive impact on the Na/K ratio. Obviously, without the nicotine depressant action on hunger, former smokers ate more food and gain weight. Regular olive oil intake strongly predicted lower salt, higher potassium consumption, and a healthier Na/K ratio. It is a paradigm of the healthier Adriatic diet. This statement was additionally supported by the result that frequent fish consumption predicts achieved targets of daily potassium intake. SES and lifestyle were strongly associated with daily salt and potassium intake, as well as with the Na/K ratio, showing meaningful (though small) relationships which are inherent to complex human-behaviour studies where context matters. From a public-health perspective, the results highlight the need for targeted nutritional interventions for lower SES populations, focusing on salt-reduction strategies and the promotion of potassium-rich foods, as well as salt substitutes. It is essential that the population receives comprehensive education regarding both the importance of reducing salt intake and increasing potassium consumption. Addressing these dietary imbalances could play a crucial role in reducing health inequalities and the burden of diet-related chronic diseases.

## Figures and Tables

**Table 1:** Comparison of potassium, Na/K ratio and salt intakes according to the socioeconomic factors.

	Potassium (g)	Na/K	NaCl (g)	*p*						
				Potassium (g)	Na/K	NaCl (g)	Group	Potassium (g)	Na/K	NaCl (g)
Monthly Income										
<2500 HRK (N = 84) (1)				0.470	<0.001	0.004	1-5	NS	0.008	0.071
***X*** (sd)	2.9 (1.2)	3.0 (1.4)	9.7 (4.4)							
SE	0.131	0.159	0.490							
median (IQR)	2.6 (2.0–3.1)	2.8 (1.9–3.6)	9.0 (6.5–11.9)							
2500–3500 HRK (N = 78) (2)							2-5	NS	<0.001	<0.001
***X*** (sd)	2.8 (0.9)	3.1 (1.3)	10.6 (4.7)							
SE	0.109	0.156	0.535							
median (IQR)	2.6 (2.0–3.5)	2.9 (2.3–3.9)	9.9 (7.2–13.9)							
3500–5000 HRK (N = 155) (3)							3-5	NS	0.003	0.015
***X*** (sd)	2.9 (1.1)	3.0 (1.5)	9.8 (4.6)							
SE	0.092	0.126	0.218							
median (IQR)	2.7 (2.0–3.5)	2.7 (1.8–3.7)	9.2 (6.4–12.9)							
5000–10,000 HRK (N = 319) (4)							4-5	NS	0.012	NS
***X*** (sd)	2.9 (1.0)	2.7 (1.4)	9.0 (3.9)							
SE	0.059	0.080	0.218							
median (IQR)	2.7 (2.0–3.5)	2.5 (1.8–3.4)	8.4 (6.1–11.2)							
>10,000 HRK (N = 237) (5)							2-4	NS	0.012	0.006
***X*** (sd)	3.0 (1.1)	2.5 (1.3)	8.7 (4.1)							
SE	0.076	0.087	0.269							
median (IQR)	2.9 (2.1–3.8)	2.3 (1.6–3.2)	8.1 (5.6–11.6)							
Professional Qualification										
Low-Skilled Worker (N = 180) (1)				0.116	<0.001	<0.001	1-5	0.723	<0.001	<0.001
$\bar{X}$ (sd)	2.8 (1.0)	3.1 (1.3)	9.9 (4.2)							
SE	0.080	0.098	0.317				1-3	0.678	0.356	0.289
median (IQR)	2.6 (2.0–3.3)	2.9 (2.2–3.9)	9.3 (7.1–12.1)							
High-Skilled (Worker) (N = 363) (2)							1-4	0.060	<0.001	0.003
$\bar{X}$ (sd)	3.0 (1.1)	2.8 (1.3)	9.7 (4.2)							
SE	0.058	0.068	0.222				1-2	0.023	0.015	0.380
median (IQR)	2.9 (2.2–3.6)	2.7 (1.8–3.5)	9.0 (6.4–12.1)							
Medium Level of Professional Qualification (N = 20) (3)							5-3	0.804	0.001	0.001
$\bar{X}$ (sd)	2.9 (1.3)	3.3 (1.3)	11.4 (5.8)							
SE	0.303	0.301	1.307				5-4	0.142	0.840	0.091
median (IQR)	2.8 (1.9–3.4)	3.3 (2.4–4.4)	11.0 (8.1–14.9)							
Higher Professional Qualification (N = 157) (4)							5-2	0.081	<0.001	<0.001
$\bar{X}$ (sd)	3.0 (1.0)	2.6 (1.8)	8.7 (4.3)							
SE	0.087	0.145	0.349				3-4	0.650	0.002	0.015
median (IQR)	2.8 (2.1–3.7)	2.3 (1.6–3.0)	8.0 (5.2–11.6)							
High Professional Qualification (N = 153) (5)							3-2	0.634	0.056	0.151
$\bar{X}$ (sd)	2.8 (1.1)	2.5 (1.3)	7.8 (3.5)							
SE	0.093	0.111	0.290				4-2	0.985	0.001	0.010
median (IQR)	2.8 (2.0–3.3)	2.2 (1.5–3.1)	7.5 (4.9–10.1)							
Educational Level										
Without Formal Education (N = 7) (1)				0.127	<0.001	0.136	2-4	0.023	<0.001	0.018
$\bar{X}$ (sd)	2.9 (1.3)	3.1 (1.3)	10.4 (4.0)							
SE	0.494	0.518	1.513							
median (IQR)	2.6 (1.8–4.4)	2.6 (2.5–2.8)	9.3 (6.5–14.4)							
<4 Years (N = 13) (2)										
$\bar{X}$ (sd)	2.8 (0.7)	2.7 (0.9)	8.9 (3.2)							
SE	0.213	0.931	0.907				3-4	NS	0.048	0.006
median (IQR)	2.7 (2.0–3.5)	2.5 (2.0–3.4)	8.1 (5.9–11.1)							
4–8 Years (N = 130) (3)										
$\bar{X}$ (sd)	2.7 (1.0)	3.2 (1.3)	9.8 (4.4)							
SE	0.092	0.122	0.393							
median (IQR)	2.5 (1.9–3.2)	2.97 (2.2–4.1)	8.7 (6.7–12.2)							
>12 Years (N = 490) (4)							2-3	0.010	<0.001	NS
$\bar{X}$ (sd)	2.9 (1.1)	2.8 (1.4)	9.4 (4.4)							
SE	0.051	0.067	0.198							
median (IQR)	2.8 (2.2–3.5)	2.8 (1.8–3.5)	8.6 (6.1–12.0)							

Note: N—total number of subjects; $\bar{X}$—mean value; sd—standard deviation; SE—standard error; IQR—interquartile range; *p*—level of statistical significance; Na/K—urinary sodium-to-potassium excretion ratio; NaCl—sodium chloride (table salt); g—gram; HRK—Croatian kuna (official currency of the Republic of Croatia until 31 December 2022); NS—not significant.

**Table 2:** Comparison of potassium, Na/K ratio and salt intakes according to the lifestyle habits (smoking status, alcohol consumption, physical activity).

	Potassium (g)	Na/K	NaCl (g)	*p*						
				Potassium (g)	Na/K	NaCl (g)	Group	Potassium (g)	Na/K	NaCl (g)
Smoking Status										
Non-Smokers (N = 463) (1)				0.004	0.449	0.041	3-1	0.078	0.293	0.047
$\bar{X}$ (sd)	2.9 (1.1)	2.8 (1.3)	9.4 (4.3)							
SE	0.051	0.060	0.201							
median (IQR)	2.8 (2.1–3.5)	2.6 (1.8–3.5)	8.6 (6.2–11.9)							
Former Smokers (N = 200) (2)							3-2	<0.001	0.971	0.015
$\bar{X}$ (sd)	3.1 (1.2)	2.9 (1.8)	9.7 (4.3)							
SE	0.087	0.131	0.310							
median (IQR)	2.9 (2.2–3.9)	2.5 (1.7–3.5)	9.3 (6.5–12.6)							
Smokers (N = 210) (3)							1-2	0.033	0.321	0.381
$\bar{X}$ (sd)	2.7 (0.9)	2.7 (1.2)	8.6 (3.9)							
SE	0.067	0.087	0.273							
median (IQR)	2.6 (2.0–3.3)	2.5 (1.9–3.2)	8.1 (5.9–10.9)							
	**Potassium (g)**	**Na/K**	**NaCl (g)**	***p***						
				**Potassium (g)**	**Na/K**	**NaCl (g)**	**Group**	**Potassium (g)**	**Na/K**	**NaCl (g)**
Alcohol Consumption										
Often (N = 36) (1)				<0.001	0.523	0.456	5-4	0.247	0.511	0.784
$\bar{X}$ (sd)	3.1 (1.3)	2.7 (1.8)	9.4 (4.6)							
SE	0.219	0.315	0.782				5-3	0.334	0.957	0.287
median (IQR)	2.9 (2.3–3.8)	2.2 (1.6–3.2)	8.1 (5.0–13.6)							
Rarely (N = 193) (2)							5-1	0.104	0.203	0.859
$\bar{X}$ (sd)	3.2 (1.2)	2.7 (1.4)	9.8 (4.5)							
SE	0.092	0.106	0.327				5-2	<0.001	0.179	0.085
median (IQR)	3.2 (2.2–3.8)	2.5 (1.7–3.5)	9.0 (6.4–12.7)							
Moderately (N = 94) (3)							4-3	0.922	0.606	0.416
$\bar{X}$ (sd)	2.8 (1.0)	3.0 (1.9)	9.6 (4.2)							
SE	0.105	0.197	0.443				4-1	0.305	0.356	0.968
median (IQR)	2.8 (2.2–3.5)	2.6 (1.8–3.5)	8.8 (6.3–13.1)							
Occasionally (N = 218) (4)							4-2	0.003	0.515	0.182
$\bar{X}$ (sd)	2.8 (0.9)	2.7 (1.2)	9.1 (3.9)							
SE	0.066	0.081	0.264				3-1	0.379	0.241	0.634
median (IQR)	2.8 (2.2–3.3)	2.5 (1.8–3.5)	8.6 (6.0–11.3)							
Never (N = 331) (5)							3-2	0.027	0.309	0.802
$\bar{X}$ (sd)	2.8 (1.0)	2.8 (1.3)	9.0 (4.2)							
SE	0.058	0.074	0.235				1-2	0.561	0.575	0.492
median (IQR)	2.6 (2.0–3.3)	2.6 (1.9–3.5)	8.4 (6.2–11.3)							
	**Potassium (g)**	**Na/K**	**NaCl (g)**	***p***						
				**Potassium (g)**	**Na/K**	**NaCl (g)**	**Group**	**Potassium (g)**	**Na/K**	**NaCl (g)**
Physical Activity										
1X Week (N = 60) (1)				0.012	0.075	0.384	5-6	0.282	0.984	0.157
$\bar{X}$ (sd)	3.0 (10)	2.8 (1.3)	9.3 (3.6)				5-7	0.661	0.257	0.506
SE	0.140	0.174	0.469							
median (IQR)	2.9 (2.0–3.8)	2.5 (1.8–3.7)	8.4 (6.5–11.8)				5-2	0.043	0.033	0.649
2X Week (N = 86) (2)							5-1	0.039	0.452	0.469
$\bar{X}$ (sd)	3.0 (1.1)	2.5 (1.0)	8.7 (3.7)				5-4	0.023	0.015	0.531
SE	0.118	0.115	0.404							
median (IQR)	2.8 (2.7–3.7)	2.5 (1.6–3.2)	8.3 (5.6–11.5)				5-3	<0.001	0.083	0.158
More than 2X Week (N = 243) (3)							6-7	0.868	0.264	0.312
$\bar{X}$ (sd)	3.1 (1.1)	2.7 (1.4)	9.6 (4.3)				6-2	0.309	0.055	0.137
SE	0.075	0.090	0.281							
median (IQR)	2.9 (2.2–3.9)	2.5 (1.7–3.5)	9.2 (6.0–12.6)				6-1	0.237	0.495	0.777
Actively Involved in Sport (N = 67) (4)							6-4	0.180	0.026	0.117
$\bar{X}$ (sd)	3.0 (1.0)	2.5 (1.3)	8.7 (3.8)				6-3	0.051	0.153	0.832
SE	0.128	0.128	0.475							
median (IQR)	3.1 (2.1–3.7)	3.1 (2.1–3.7)	8.0 (5.5–10.6)				7-2	0.869	0.630	0.606
Occasionally (N = 270) (5)							7-1	0.792	0.400	0.378
$\bar{X}$ (sd)	2.7 (1.0)	2.9 (1.5)	9.1 (4.5)				7-4	0.761	0.750	0.658
SE	0.062	0.094	0.274							
median (IQR)	2.6 (2.0–3.3)	2.7 (1.9–3.6)	8.4 (6.2–11.2)				7-3	0.741	0.446	0.334
Never (N = 141) (6)							2-1	0.797	0.350	0.342
$\bar{X}$ (sd)	2.9 (1.2)	2.9 (1.4)	9.6 (4.2)				2-4	0.713	0.678	0.858
SE	0.101	0.125	0.361							
median (IQR)	2.7 (2.1–3.4)	2.7 (1.9–3.6)	8.7 (6.7–12.4)				2-3	0.593	0.376	0.149
Don’t Want to Answer (N = 6) (7)							1-4	0.925	0.206	0.288
$\bar{X}$ (sd)	2.8 (0.7)	2.3 (1.2)	8.4 (6.0)				1-3	0.868	0.749	0.883
SE	0.311	0.524	2.463							
median (IQR)	3.2 (2.0–3.3)	2.0 (1.4–3.4)	6.5 (3.8–14.1)				4-3	0.958	0.196	0.128

**Table 3:** Comparison of potassium, Na/K ratio and salt intakes according to the lifestyle habits (consumption of processed meat, red meat, poultry, fish, and olive oil).

	Potassium (g)	Na/K	NaCl (g)	*p*						
				Potassium (g)	Na/K	NaCl (g)	Group	Potassium (g)	Na/K	NaCl (g)
Processed Meat										
Not at All (N = 74) (1)				0.092	0.004	<0.001	1-5	0.476	0.781	0.664
$\bar{X}$ (sd)	2.6 (1.0)	2.4 (1.2)	7.2 (3.3)							
SE	0.117	0.144	0.395				1-3	0.018	0.006	<0.001
median (IQR)	2.5 (1.9–3.2)	2.2 (1.4–3.1)	6.7 (4.5–8.6)							
Very Rarely (Several Times Per Month) (N = 336) (2)							1-2	0.015	0.263	<0.001
$\bar{X}$ (sd)	2.9 (1.1)	2.7 (1.3)	8.8 (3.7)							
SE	0.061	0.075	0.205				1-4	0.006	0.007	<0.001
median (IQR)	2.8 (2.1–3.6)	2.4 (1.7–3.2)	8.4 (6.0–11.2)							
2X Per Week (N = 301) (3)							5-3	0.992	0.252	0.284
$\bar{X}$ (sd)	2.9 (1.1)	2.9 (1.5)	9.8 (4.6)							
SE	0.066	0.088	0.268				5-2	0.980	0.525	0.558
median (IQR)	2.7 (2.1–3.5)	2.8 (1.9–3.6)	9.1 (6.4–12.5)							
Every Day/Almost Every Day (N = 156) (4)							5-4	0.833	0.229	0.196
$\bar{X}$ (sd)	3.0 (1.0)	3.0 (1.4)	10.2 (4.4)							
SE	0.086	0.115	0.356				3-2	0.937	0.008	0.011
median (IQR)	2.9 (2.2–3.7)	2.7 (2.0–3.5)	9.6 (7.1–13.3)							
Don’t Want to Answer (N = 6) (5)							3-4	0.397	0.776	0.330
$\bar{X}$ (sd)	2.8 (0.7)	2.3 (1.2)	8.4 (6.0)							
SE	0.311	0.524	2.463				2-4	0.425	0.014	0.002
median (IQR)	3.2 (2.0–3.3)	2.0 (1.4–3.4)	6.5 (3.8–14.1)							
	**Potassium (g)**	**Na/K**	**NaCl (g)**	***p***						
				**Potassium (g)**	**Na/K**	**NaCl (g)**	**Group**	**Potassium (g)**	**Na/K**	**NaCl (g)**
Red Meat										
Not at All (N = 21) (1)				0.039	0.055	0.063	2-3	0.159	0.557	0.909
$\bar{X}$ (sd)	3.1 (1.3)	2.0 (1.1)	7.2 (3.6)							
SE	0.293	0.253	0.802				2-5	0.675	0.290	0.457
median (IQR)	3.2 (2.4–3.5)	1.9 (1.1–2.9)	6.5 (4.7–8.6)							
Very Rarely (Several Times Per Month) (N = 130) (2)							2-4	0.003	0.329	0.380
$\bar{X}$ (sd)	2.7 (1.1)	3.0 (1.5)	9.2 (4.1)							
SE	0.096	0.137	0.365				2-1	0.133	0.005	0.019
median (IQR)	2.5 (1.9–3.4)	2.6 (2.0–3.4)	8.4 (6.0–12.1)							
2X Per Week (N = 495) (3)							3-5	0.930	0.350	0.434
$\bar{X}$ (sd)	2.9 (1.0)	2.8 (1.4)	9.2 (4.1)							
SE	0.049	0.063	0.186				3-4	0.023	0.537	0.289
median (IQR)	2.7 (2.1–3.4)	2.6 (1.8–3.5)	8.5 (6.3–11.6)							
Every Day/Almost Every Day (N = 221) (4)							3-1	0.336	0.006	0.011
$\bar{X}$ (sd)	3.1 (1.1)	2.8 (1.4)	9.7 (4.5)							
SE	0.076	0.096	0.307				5-4	0.720	0.419	0.325
median (IQR)	2.9 (2.2–3.7)	2.5 (1.8–3.4)	9.0 (6.2–12.6)							
Don’t Want to Answer (N = 6) (5)							5-1	0.700	0.627	0.601
$\bar{X}$ (sd)	2.8 (0.7)	2.3 (1.2)	8.4 (6.0)							
SE	0.311	0.524	2.463				4-1	0.894	0.014	0.004
median (IQR)	3.2 (2.0–3.3)	2.0 (1.4–3.4)	6.5 (3.8–14.1)							
	**Potassium (g)**	**Na/K**	**NaCl (g)**	***p***						
				**Potassium (g)**	**Na/K**	**NaCl (g)**	**Group**	**Potassium (g)**	**Na/K**	**NaCl (g)**
Fish										
Not at All (N = 38) (1)				0.768	<0.001	<0.001	2-5	0.898	0.148	0.193
$\bar{X}$ (sd)	2.9 (0.8)	2.7 (1.1)	8.9 (3.2)							
SE	0.134	0.193	0.525				2-3	0.237	<0.001	<0.001
median (IQR)	3.1 (2.2–3.4)	2.5 (1.9–3.2)	8.5 (6.0–11.0)							
Very Rarely (Several Times Per Month) (N = 396) (2)							2-1	0.532	0.104	0.160
$\bar{X}$ (sd)	2.8 (1.0)	3.0 (1.3)	10.1 (4.2)							
SE	0.051	0.065	0.215				2-4	0.465	0.012	0.069
median (IQR)	2.7 (2.1–3.4)	2.8 (2.0–3.8)	9.4 (6.9–13.0)							
2X Per Week (N = 410) (3)							5-3	0.941	0.688	0.731
$\bar{X}$ (sd)	3.0 (1.1)	2.6 (1.5)	8.5 (4.1)							
SE	0.059	0.077	0.206				5-1	0.903	0.467	0.500
median (IQR)	2.8 (2.1–3.7)	2.3 (1.5–3.1)	8.0 (5.4–10.9)							
Every Day/Almost Every Day (N = 23) (4)							5-4	0.821	0.900	0.751
$\bar{X}$ (sd)	3.1 (1.6)	2.4 (1.3)	8.6 (4.5)							
SE	0.336	0.272	0.957				3-1	0.892	0.363	0.360
median (IQR)	3.2 (1.6–4.2)	2.1 (1.9–2.8)	7.6 (4.4–11.8)							
Don’t Want to Answer (N = 6) (5)							3-4	0.731	0.616	0.985
$\bar{X}$ (sd)	2.8 (0.7)	2.3 (1.2)	8.4 (6.0)							
SE	0.311	0.524	2.463				1-4	0.848	0.322	0.567
median (IQR)	3.2 (20–3.3)	2.0 (1.4–3.4)	6.5 (3.8–14.1)							
	**Potassium (g)**	**Na/K**	**NaCl (g)**	***p***						
				**Potassium (g)**	**Na/K**	**NaCl (g)**	**Group**	**Potassium (g)**	**Na/K**	**NaCl (g)**
Polutry										
Not at All (N = 17) (1)				0.295	0.017	<0.001	2-4	0.756	0.311	0.309
$\bar{X}$ (sd)	3.1 (1.5)	2.1 (1.2)	7.4 (3.9)							
SE	0.364	0.297	0.947				2-5	0.744	0.363	0.486
median (IQR)	2.9 (2.0–3.9)	2.1 (1.2–2.9)	6.2 (4.4–10.6)							
Very Rarely (Several Times Per Month) (N = 57) (2)							2-3	0.170	0.726	0.333
$\bar{X}$ (sd)	2.8 (1.2)	2.8 (1.3)	9.1 (4.0)							
SE	0.161	0.184	0.539				2-1	0.428	0.055	0.838
median (IQR)	2.7 (1.9–3.6)	2.6 (1.8–3.4)	9.0 (5.9–11.9)							
2X Per Week (N = 533) (3)							4-5	0.819	0.558	0.716
$\bar{X}$ (sd)	3.0 (1.0)	2.8 (1.3)	9.7 (4.2)							
SE	0.047	0.057	0.183				4-3	0.054	0.009	<0.001
median (IQR)	2.8 (2.1–3.6)	2.6 (1.9–3.5)	9.1 (6.5–12.0)							
Every Day/Almost Every Day (N = 260) (4)							4-1	0.488	0.127	0.189
$\bar{X}$ (sd)	2.8 (1.1)	2.7 (1.6)	8.6 (4.2)							
SE	0.068	0.104	0.266				5-3	0.901	0.285	0.290
median (IQR)	2.7 (2.1–3.3)	2.5 (1.6–3.4)	7.9 (5.5–10.4)							
Don’t Want to Answer (N = 6) (5)							5-1	0.868	0.768	0.707
$\bar{X}$ (sd)	2.8 (0.7)	2.3 (1.2)	8.4 (6.0)							
SE	0.311	0.524	2.463				3-1	0.909	0.019	0.129
median (IQR)	3.2 (2.0–3.3)	2.0 (1.4–3.4)	6.5 (3.8–14.1)							
	**Potassium (g)**	**Na/K**	**NaCl (g)**	***p***						
				**Potassium (g)**	**Na/K**	**NaCl (g)**	**Group**	**Potassium (g)**	**Na/K**	**NaCl (g)**
Olive Oil										
First Choice (N = 194) (1)				0.290	<0.001	<0.001	1-2	0.237	0.136	0.212
$\bar{X}$ (sd)	3.0 (1.1)	2.5 (1.4)	8.3 (3.8)							
SE	0.085	0.105	0.277				1-3	0.018	<0.001	<0.001
median (IQR)	2.9 (2.1–3.7)	2.1 (1.5–3.0)	8.0 (5.3–10.5)							
Second Choice (N = 182) (2)							1-4	0.021	<0.001	<0.001
$\bar{X}$ (sd)	3.0 (1.2)	2.6 (1.4)	8.9 (4.2)							
SE	0.092	0.109	0.313							
median (IQR)	2.9 (2.1–3.9)	2.4 (1.7–3.1)	8.4 (5.7–11.8)							
Third Choice (N = 131) (3)							2-3	<0.001	<0.001	0.002
$\bar{X}$ (sd)	2.8 (0.9)	3.2 (1.4)	10.6 (4.8)							
SE	0.084	0.127	0.425				2-4	<0.001	<0.001	<0.001
median (IQR)	2.7 (2.2–3.4)	2.9 (2.1–4.0)	9.4 (7.2–13.4)							
Fourth Choice (N = 150) (4)							3-4	0.286	0.131	0.182
$\bar{X}$ (sd)	2.8 (0.9)	3.3 (1.1)	10.9 (4.0)							
SE	0.077	0.092	0.329							
median (IQR)	2.7 (2.1–3.3)	3.2 (2.5–4.0)	10.6 (8.0–13.4)							

Note: N—total number of subjects; $\bar{X}$—mean value; sd—standard deviation; SE—standard error; IQR—interquartile range; *p*—level of statistical significance; Na/K—urinary sodium-to-potassium excretion ratio; NaCl—sodium chloride (table salt); g—gram.

**Table 4:** Linear regression model for daily salt intake.

	B	SE	β	*p*
Constant	5.967	1.222		<0.001
Education	0.553	0.324	0.094	0.089
Qualification	−0.559	0.176	−0.182	0.002
Processed meat	0.828	0.190	0.168	<0.001
Olive oil	0.620	0.159	0.161	<0.001
Smoking	−0.488	0.199	−0.092	0.015

**Table 5:** Linear regression model for daily potassium intake.

	B	SE	β	p
Constant	2.241	0.397		<0.001
Education	0.155	0.087	0.102	0.077
Qualification	−0.110	0.047	−0.140	0.019
Processed meat	0.130	0.051	0.103	0.010
Olive oil	−0.117	0.043	−0.118	0.006
Smoking	−0.143	0.053	−0.105	0.007
Salad	0.158	0.072	0.086	0.029

**Table 6:** Multinominal regression for recommended daily salt intake (<5 g/day).

	B	SE	*p*	Exp (B)	95% CI
Intercept	1.393	0.570	0.014		
Smoking status	−0.303	0.144	0.035	0.739	0.558–0.979
Processed meat	0.397	0.145	0.006	1.487	1.119–1.977
Olive oil	0.392	0.131	0.003	1.480	1.145–1.913
Qualification	−0.282	0.087	0.001	0.754	0.636–0.894

**Table 7:** Multinominal regression for recommended potassium intake (3.5 g per day).

	B	SE	*p*	Exp (B)	95% CI
Intercept	−2.833	0.839	<0.001		
Fish consumption	0.438	0.257	0.089	1.549	0.936–2.564
Olive oil	−0.293	0.145	0.044	0.746	0.562–0.992

## Data Availability

The original contributions presented in this study are included in the article/Supplementary Material. Further inquiries can be directed to the corresponding authors.

## Supplementary Materials

The following supporting information can be downloaded at: https://www.mdpi.com/article/10.3390/nu18040615/s1, Figure S1: Spearman rank correlations between socioeconomic factors and salt intake and. sodium–to–potassium ratio, adjusted for age and gender; Figure S2: Heat map of daily salt and potassium intake and sodium-to-potassium ratio categories according to the socioeconomic factors; Figure S3: Spearman rank correlations between dietary habits and salt intake and sodium–to–potassium ratio, adjusted for age and gender; Figure S4: Heat maps of daily salt and potassium intake and sodium-to-potassium ratio categories according to the dietary habits; Table S1: Relationship of risk factors with salt and potassium intake, and sodium-to-potassium ratio.

## References

[1] Egan B.M., Lackland D.T., Sutherland S.E., Rakotz M.K., Williams J., Commodore-Mensah Y., Jones D.W., Kjeldsen S., Campbell N.R.C., Parati G. (2025). PERSPECTIVE–The Growing Global Benefits of Limiting Salt Intake: An urgent call from the World Hypertension League for more effective policy and public health initiatives. J. Hum. Hypertens..

[2] Han F., Li W., Duan N., Hu X., Yao N., Yu G., Qu J. (2025). Relationship between salt intake and cardiovascular disease. J. Clin. Hypertens..

[3] Mozaffarian D., Fahimi S., Singh G.M., Micha R., Khatibzadeh S., Engell R.E., Lim S., Danaei G., Ezzati M., Powles J. (2014). Global Sodium Consumption and Death from Cardiovascular Causes. N. Engl. J. Med..

[4] Osoro I., Rajanandh M.G. (2025). A comprehensive review on cardiovascular disorders development due to salt intake: An emphasis on policy implementation. Health Res. Policy Syst..

[5] Wu Q., Burley G., Li L., Lin S., Shi Y. (2023). The role of dietary salt in metabolism and energy balance: Insights beyond cardiovascular disease. Diabetes Obes. Metab..

[6] He F.J., MacGregor G.A. (2014). Reducing Population Salt Intake—Time for Global Action. J. Clin. Hypertens..

[7] Du X., Zhu Y., Guo J., Chen X., Zhang J., Lu F., Xu C., Liang M., Wang M., Zhong J. (2025). Effect of salt reduction interventions in lowering blood pressure and salt intake in Zhejiang Province, China, 2017–2021: A randomized controlled trial. Nutrients.

[8] Okuda N., Stamler J., Brown I.J., Ueshima H., Miura K., Okayama A., Saitoh S., Nakagawa H., Sakata K., Yoshita K. (2014). Individual efforts to reduce salt intake in China, Japan, UK, USA. J. Hypertens..

[9] World Health Organization: WHO Sodium Reduction. https://www.who.int/news-room/fact-sheets/detail/sodium-reduction.

[10] Kwong E.J.L., Whiting S., Bunge A.C., Leven Y., Breda J., Rakovac I., Cappuccio F.P., Wickramasinghe K. (2022). Population-level salt intake in the WHO European Region in 2022: A systematic review. Public Health Nutr..

[11] Opasanant P., Sukwong P. (2023). Formation and implementation of public health policy toward salt reduction in food consumption. J. Prim. Care Community Health.

[12] Reddin C., Ferguson J., Murphy R., Clarke A., Judge C., Griffith V., Alvarez A., Smyth A., Mente A., Yusuf S. (2023). Global mean potassium intake: A systematic review and Bayesian meta-analysis. Eur. J. Nutr..

[13] Kumssa D.B., Joy E.J.M., Broadley M.R. (2021). Global Trends (1961–2017) in human dietary potassium supplies. Nutrients.

[14] Mancia G., Kreutz R., Brunström M. (2023). 2023 ESH Guidelines for the management of arterial hypertension The Task Force for the management of arterial hypertension of the European Society of Hypertension. J. Hypertens..

[15] McEvoy J.W., McCarthy C.P., Bruno R.M., Brouwers S., Canavan M.D., Ceconi C., Christodorescu R.M., Daskalopoulou S.S., Ferro C.J., Gerdts E. (2024). 2024 ESC Guidelines for the management of elevated blood pressure and hypertension: Developed by the task force on the management of elevated blood pressure and hypertension of the European Society of Cardiology (ESC) and endorsed by the European Society of Endocrinology (ESE) and the European Stroke Organisation (ESO). Eur. Heart J..

[16] Bhagavathula A.S., Refaat S.A., Bentley B.L., Rahmani J. (2021). Association between intake of sodium, potassium, sodium-to-potassium ratio, and blood pressure among US adults. Int. J. Vitam. Nutr. Res..

[17] Perez V., Chang E.T. (2014). Sodium-to-Potassium ratio and blood pressure, hypertension, and related factors. Adv. Nutr..

[18] Du X., Fang L., Xu J., Chen X., Bai Y., Wu J., Wu L., Zhong J. (2022). The association of knowledge, attitudes and behaviors related to salt with 24-h urinary sodium, potassium excretion and hypertensive status. Sci. Rep..

[19] Park S.K., Oh C., Ryoo J., Jung J.Y. (2025). Intake of sodium and potassium, sodium-potassium intake ratio, and their relation to the risk of diabetes mellitus. Sci. Rep..

[20] De Mestral C., Mayén A., Petrovic D., Marques-Vidal P., Bochud M., Stringhini S. (2017). Socioeconomic Determinants of Sodium Intake in Adult Populations of High-Income Countries: A Systematic Review and Meta-Analysis. Am. J. Public Health.

[21] Miyagawa N., Okuda N., Nakagawa H., Takezaki T., Nishi N., Takashima N., Miura K. (2018). Socioeconomic status associated with urinary sodium and potassium excretion in Japan: NIPPON DATA2010. J. Epidemiol..

[22] Darmon N., Drewnowski A. (2008). Does social class predict diet quality?. Am. J. Clin. Nutr..

[23] Shohaimi S., Bingham S., Welch A., Luben R., Day N., Wareham N., Khaw K. (2004). Occupational social class, educational level and area deprivation independently predict plasma ascorbic acid concentration: A cross-sectional population based study in the Norfolk cohort of the European Prospective Investigation into Cancer (EPIC-Norfolk). Eur. J. Clin. Nutr..

[24] Tian H.G., Hu G.A.N.G., Dong Q.N., Yang X.L., Nan Y., Pietinen P., Nissinen A.U.L.I.K.K.I. (1996). Dietary sodium and potassium, socioeconomic status and blood pressure in a Chinese population. Appetite.

[25] Drewnowski A., Specter S. (2004). Poverty and obesity: The role of energy density and energy costs. Am. J. Clin. Nutr..

[26] Stringhini S., Carmeli C., Jokela M., Avendaño M., Muennig P., Guida F., Zins M. (2017). Socioeconomic status and the 25 × 25 risk factors as determinants of premature mortality: A multicohort study and meta-analysis of 1·7 million men and women. Lancet.

[27] Dubowitz T., Heron M., Bird C.E., Lurie N., Finch B.K., Basurto-Dávila R., Hale L., Escarce J.J. (2008). Neighborhood socioeconomic status and fruit and vegetable intake among whites, blacks, and Mexican Americans in the United States. Am. J. Clin. Nutr..

[28] Murakami K., Sasaki S., Takahashi Y., Uenishi K. (2009). Neighborhood Socioeconomic Disadvantage Is Associated with Higher Ratio of 24-Hour Urinary Sodium to Potassium in Young Japanese Women. J. Am. Diet. Assoc..

[29] Vaudin A., Wambogo E., Moshfegh A.J., Sahyoun N.R. (2021). Sodium and potassium intake, the sodium to potassium ratio, and associated characteristics in older adults, NHANES 2011–2016. J. Acad. Nutr. Diet..

[30] Nichols S., Dalrymple N., Prout P., Ramcharitar-Bourne A. (2021). Socio-demographic factors in relation to habitual sodium and potassium intakes among adults in Trinidad and Tobago. Nutr. Health.

[31] Yi S.S., Curtis C.J., Angell S.Y., Anderson C.A., Jung M., Kansagra S.M. (2014). Highlighting the ratio of sodium to potassium in population-level dietary assessments: Cross-sectional data from New York City, USA. Public Health Nutr..

[32] Hu N., McLean R. (2025). Lowering sodium intake: Reduction and substitution for cardiovascular health. Int. J. Vitam. Nutr. Res..

[33] Yamashita M., Tabara Y., Higo Y., Setoh K., Kawaguchi T., Takahashi Y., Kosugi S., Nakayama T., Matsuda F., Wakamura T. (2018). Association between socioeconomic factors and urinary sodium-to-potassium ratio: The Nagahama Study. Hypertens. Res..

[34] D’Elia L., Brajović M., Klisic A., Breda J., Jewell J., Cadjenović V., Cappuccio F.P. (2019). Sodium and potassium intake, knowledge attitudes and behaviour towards salt consumption amongst adults in Podgorica, Montenegro. Nutrients.

[35] Hjartåker A., Lund E. (1998). Relationship between dietary habits, age, lifestyle, and socio-economic status among adult Norwegian women. The Norwegian Women and Cancer Study. Eur. J. Clin. Nutr..

[36] Fiorito G., McCrory C., Robinson O., Carmeli C., Rosales C.O., Zhang Y., Colicino E., Dugué P., Artaud F., McKay G.J. (2019). Socioeconomic position, lifestyle habits and biomarkers of epigenetic aging: A multi-cohort analysis. Aging.

[37] Stringhini S. (2010). Association of socioeconomic position with health behaviors and mortality. JAMA.

[38] Beilin L.J. (1999). Lifestyle and Hypertension—An Overview. Clin. Exp. Hypertens..

[39] Trichopoulou A. (2004). Traditional Mediterranean diet and longevity in the elderly: A review. Public Health Nutr..

[40] Filippou C., Tatakis F., Polyzos D., Manta E., Thomopoulos C., Nihoyannopoulos P., Tousoulis D., Tsioufis K. (2022). Overview of salt restriction in the Dietary Approaches to Stop Hypertension (DASH) and the Mediterranean diet for blood pressure reduction. Rev. Cardiovasc. Med..

[41] Bach-Faig A., Berry E.M., Lairon D., Reguant J., Trichopoulou A., Dernini S., Medina F.X., Battino M., Belahsen R., Miranda G. (2011). Mediterranean diet pyramid today. Science and cultural updates. Public Health Nutr..

[42] Glavić M.M., Bilajac L., Bolješić M., Bubaš M., Capak K., Domislović M., Džakula A., Fuček M., Gellineo L., Jelaković A. (2024). Assessment of Salt, Potassium, and Iodine Intake in the Croatian Adult Population Using 24 h Urinary Collection: The EH-UH 2 Study. Nutrients.

[43] Jelaković B., Glavić M.M., Sermek M.B., Bilajac L., Bubaš M., Služek V.B., Capak K., Drenjančević I., Bošković A.G., Jelaković A. (2024). Croatian Action on Salt and Health (CRASH): On The Road To Success—Less Salt, More Health. Nutrients.

[44] Jelaković A., Radunović D., Josipović J., Vrkić T.Ž., Gellineo L., Domislović M., Prelević V., Živko M., Fuček M., Glavić M.M. (2024). PREVALENCE, characteristics, and awareness of chronic kidney disease in Croatia: The EH-UH 2 study. J. Clin. Med..

[45] World Medical Association (2008). Declaration of Helsinki. Recommendations guiding doctors in clinical research. Bull. World Health Org..

[46] Cappuccio F.P., Ji C., Donfrancesco C., Palmieri L., Ippolito R., Vanuzzo D., Giampaoli S., Strazzullo P. (2015). Geographic and socioeconomic variation of sodium and potassium intake in Italy: Results from the MINISAL-GIRCSI programme. BMJ Open.

[47] Navarro A.C., Gallagher K., Griffin S., Leydon C.L., Perry I.J., Harrington J.M. (2024). Systematic Review on the impact of Salt-Reduction initiatives by Socioeconomic Position to address health inequalities in adult populations. Nutr. Rev..

[48] Drewnowski A., Rehm C.D., Maillot M., Monsivais P. (2014). The relation of potassium and sodium intakes to diet cost among US adults. J. Hum. Hypertens..

[49] Baer D.J., Althouse A., Hermann M., Johnson J., Maki K.C., Marklund M., Vogt L., Wesson D., Stallings V.A. (2021). Targeting the Dietary NA:K Ratio—Considerations for design of an intervention study to impact blood pressure. Adv. Nutr..

[50] Dyer A., Elliott P., Stamler J., Chan Q., Ueshima H., Zhou B. (2003). Dietary intake in male and female smokers, ex-smokers, and never smokers: The INTERMAP Study. J. Hum. Hypertens..

[51] van den Berg E.H., Gruppen E.G., Blokzijl H., Bakker S.J.L., Dullaart R.P.F. (2019). Higher Sodium Intake Assessed by 24 Hour Urinary Sodium Excretion Is Associated with Non-Alcoholic Fatty Liver Disease: The PREVEND Cohort Study. J. Clin. Med..

[52] Gholami A., Ramezani A.M., Heshmati M., Hariri M. (2025). Factors related to high sodium intake based on 24-hour urinary sodium excretion in population aged 50 years and older. Sci. Rep..

[53] Baek S., Kim H. (2023). Association of Dietary Sodium-to-Potassium Ratio with Cardiometabolic Risk Factors in Korean Adults: Findings from the Korean National Health and Nutrition Examination Survey. Nutrients.

[54] Kwon Y., Lee H.S., Park G., Lee J. (2022). Association between dietary sodium, potassium, and the sodium-to-potassium ratio and mortality: A 10-year analysis. Front. Nutr..

[55] Ndanuko R.N., Ibrahim R., Hapsari R.A., Neale E.P., Raubenheimer D., Charlton K.E. (2021). Association between the Urinary So-dium to Potassium Ratio and Blood Pressure in Adults: A Systematic Review and Meta-Analysis. Adv. Nutr..

[56] Assessment of Sodium and Potassium in Processed Foods in An Urban Area in China. PubMed. https://pubmed.ncbi.nlm.nih.gov/7796788/.

[57] Malavolti M., Naska A., Fairweather-Tait S.J., Malagoli C., Vescovi L., Marchesi C., Vinceti M., Filippini T. (2021). Sodium and potassium content of foods consumed in an Italian population and the impact of adherence to a Mediterranean diet on their intake. Nutrients.

[58] Mosallanezhad Z., Jalali M., Bahadoran Z., Mirmiran P., Azizi F. (2023). Dietary sodium to potassium ratio is an independent predictor of cardiovascular events: A longitudinal follow-up study. BMC Public Health.

[59] Morrissey E., Giltinan M., Kehoe L., Nugent A.P., McNulty B.A., Flynn A., Walton J. (2020). Sodium and Potassium Intakes and Their Ratio in Adults (18–90 y): Findings from the Irish National Adult Nutrition Survey. Nutrients.

[60] Petit G., Jury V., De Lamballerie M., Duranton F., Pottier L., Martin J. (2019). Salt Intake from Processed Meat Products: Benefits, Risks and Evolving Practices. Compr. Rev. Food Sci. Food Saf..

[61] Mhurchu C.N., Capelin C., Dunford E.K., Webster J.L., Neal B.C., Jebb S.A. (2010). Sodium content of processed foods in the United Kingdom: Analysis of 44,000 foods purchased by 21,000 households. Am. J. Clin. Nutr..

[62] Górska-Warsewicz H., Rejman K., Laskowski W., Kowalcze K. (2019). Food sources of potassium in the average Polish diet. Nutrients.

[63] Kaufman A., Augustson E.M., Patrick H. (2012). Unraveling the Relationship between Smoking and Weight: The Role of Sedentary Behavior. J. Obes..

[64] Al-Mawali A., D’Elia L., Jayapal S.K., Morsi M., Al-Shekaili W.N., Pinto A.D., Cappuccio F.P. (2020). National survey to estimate sodium and potassium intake and knowledge attitudes and behaviours towards salt consumption of adults in the Sultanate of Oman. BMJ Open.

[65] Song Y., Li Y., Guo C., Wang Y., Huang L., Tan M., Ma Y. (2021). Cross-sectional comparisons of sodium content in processed meat and fish products among five countries: Potential for feasible targets and reformulation. BMJ Open.

[66] Wu P., Yang S., Wong T., Chen T., Chen H., Chen T., Chen Y. (2015). Association of Processed Meat Intake with Hypertension Risk in Hemodialysis Patients: A Cross-Sectional Study. PLoS ONE.

[67] Freedman L.S., Commins J.M., Moler J.E., Willett W., Tinker L.F., Subar A.F., Prentice R.L. (2015). Pooled results from 5 validation studies of dietary Self-Report instruments using recovery biomarkers for potassium and sodium intake. Am. J. Epidemiol..

[68] Soh Y.C., Yap K.H., McGrattan A., Yasin S., Reidpath D., Siervo M., Mohan D. (2022). Protocol for a systematic review assessing the measurement of dietary sodium intake among adults with elevated blood pressure. BMJ Open.

