# Supplementary material for: Impact of Socioeconomic Factors and Lifestyle on Salt and Potassium Intake and Sodium-to-Potassium Ratio: EH-UH 2 Study
Source: Nutrients. 2026 Feb 13;18(4):615. doi: 10.3390/nu18040615 (PMC12942789; doi:10.3390/nu18040615)
Supplement: Supplementary file 1 [file nutrients-18-00615-s001.zip › nutrients-4140307-supplementary.pdf]

Supplementary Materials

3. Results

3.1. Salt and potassium intake, sodium-to-potassium ratio and socioeconomic factors

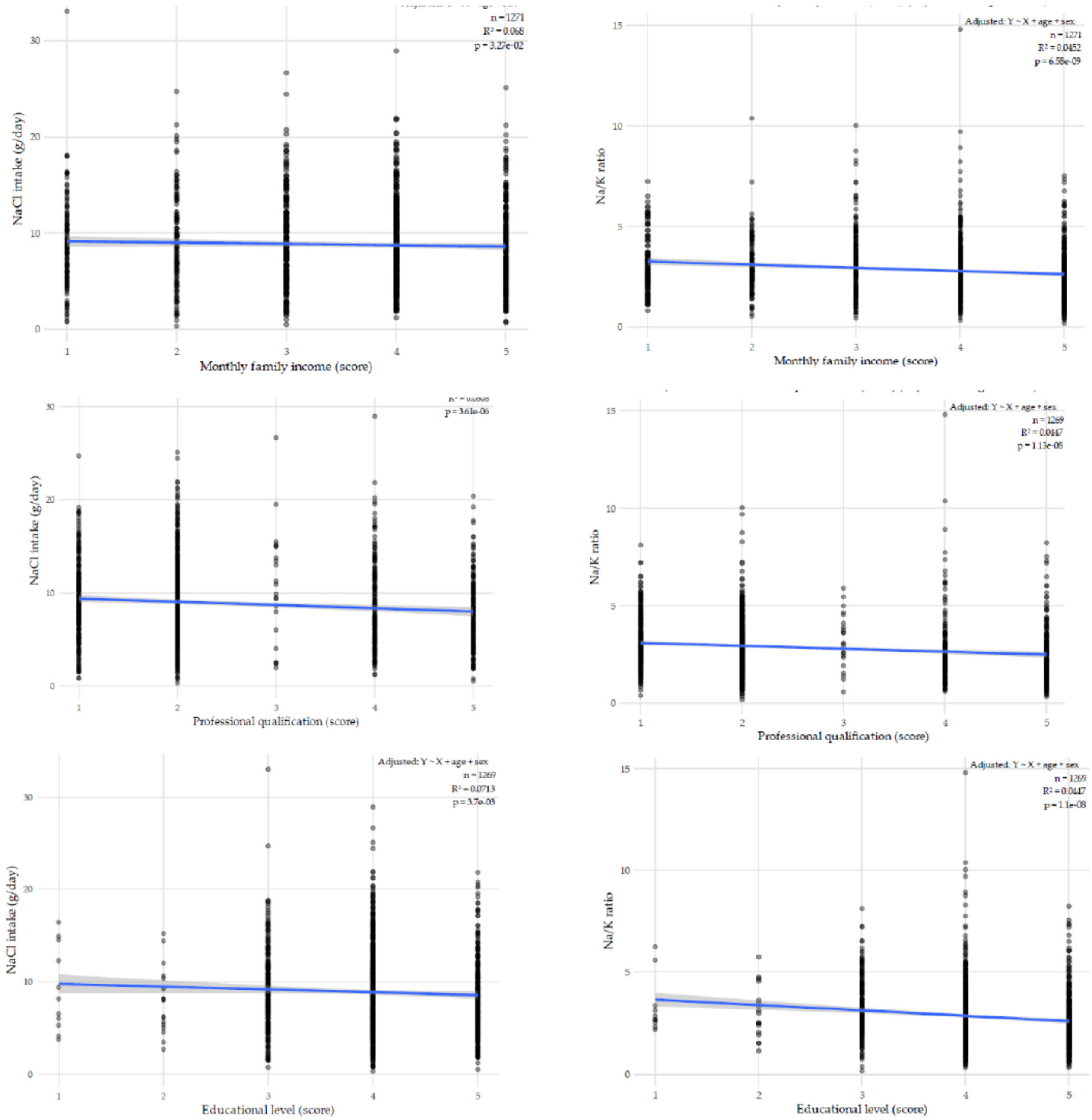

Figure S1. Spearman rank correlations between socioeconomic factors and salt intake and. sodium–to–potassium ratio, adjusted for age and gender

		Daily NaCl intake category			Daily potassium intake category			Na/K ratio category		
		< 5 g/day	5-10 g/day	> 10 g/day	< 3 g/day	3-3.5 g/day	> 3.5 g/day	< 1	1-2.5	> 2.5
Monthly family income	> 10000 HRK	72<br>23%	145<br>46%	100<br>32%	171<br>54%	57<br>18%	89<br>28%	27<br>9%	143<br>45%	147<br>46%
	5000–10000 HRK	82<br>18%	212<br>46%	165<br>36%	288<br>63%	70<br>15%	101<br>22%	19<br>4%	197<br>43%	243<br>53%
	3500–5000 HRK	45<br>19%	95<br>41%	94<br>40%	151<br>65%	33<br>14%	50<br>21%	10<br>4%	76<br>32%	148<br>63%
	2500–3500 HRK	30<br>22%	55<br>40%	51<br>38%	95<br>70%	18<br>13%	23<br>17%	5<br>4%	38<br>28%	93<br>68%
	< 2500 HRK	24<br>19%	58<br>45%	47<br>36%	90<br>70%	15<br>12%	24<br>19%	1<br>1%	49<br>38%	79<br>61%
Professional qualification	High prof. qualification	61<br>29%	96<br>46%	52<br>25%	133<br>64%	32<br>15%	44<br>21%	20<br>10%	97<br>46%	92<br>44%
	Higher prof. qualification	46<br>22%	87<br>42%	72<br>35%	115<br>56%	34<br>17%	56<br>27%	12<br>6%	99<br>48%	94<br>46%
	Medium level of prof. qual.	5<br>23%	6<br>27%	11<br>50%	13<br>59%	5<br>23%	4<br>18%	1<br>5%	6<br>27%	15<br>68%
	High-skilled worker	91<br>17%	244<br>45%	207<br>38%	322<br>59%	89<br>16%	131<br>24%	25<br>5%	210<br>39%	307<br>57%
	Low-skilled worker	50<br>17%	130<br>44%	115<br>39%	210<br>71%	33<br>11%	52<br>18%	4<br>1%	89<br>30%	202<br>68%
Educational level	> 12 years	70<br>21%	165<br>49%	99<br>30%	201<br>60%	49<br>15%	84<br>25%	21<br>6%	157<br>47%	156<br>47%
	8–12 years	139<br>20%	289<br>42%	267<br>38%	417<br>60%	116<br>17%	162<br>23%	35<br>5%	272<br>39%	388<br>56%
	4–8 years	39<br>18%	94<br>44%	82<br>38%	155<br>72%	26<br>12%	34<br>16%	6<br>3%	64<br>30%	145<br>67%
	< 4 years	3<br>17%	10<br>56%	5<br>28%	12<br>67%	2<br>11%	4<br>22%	0<br>0%	6<br>33%	12<br>67%
	No school	2<br>18%	5<br>45%	4<br>36%	8<br>73%	0<br>0%	3<br>27%	0<br>0%	2<br>18%	9<br>82%

Figure S2. Heat map of daily salt and potassium intake and sodium-to-potassium ratio categories according to the socioeconomic factors.

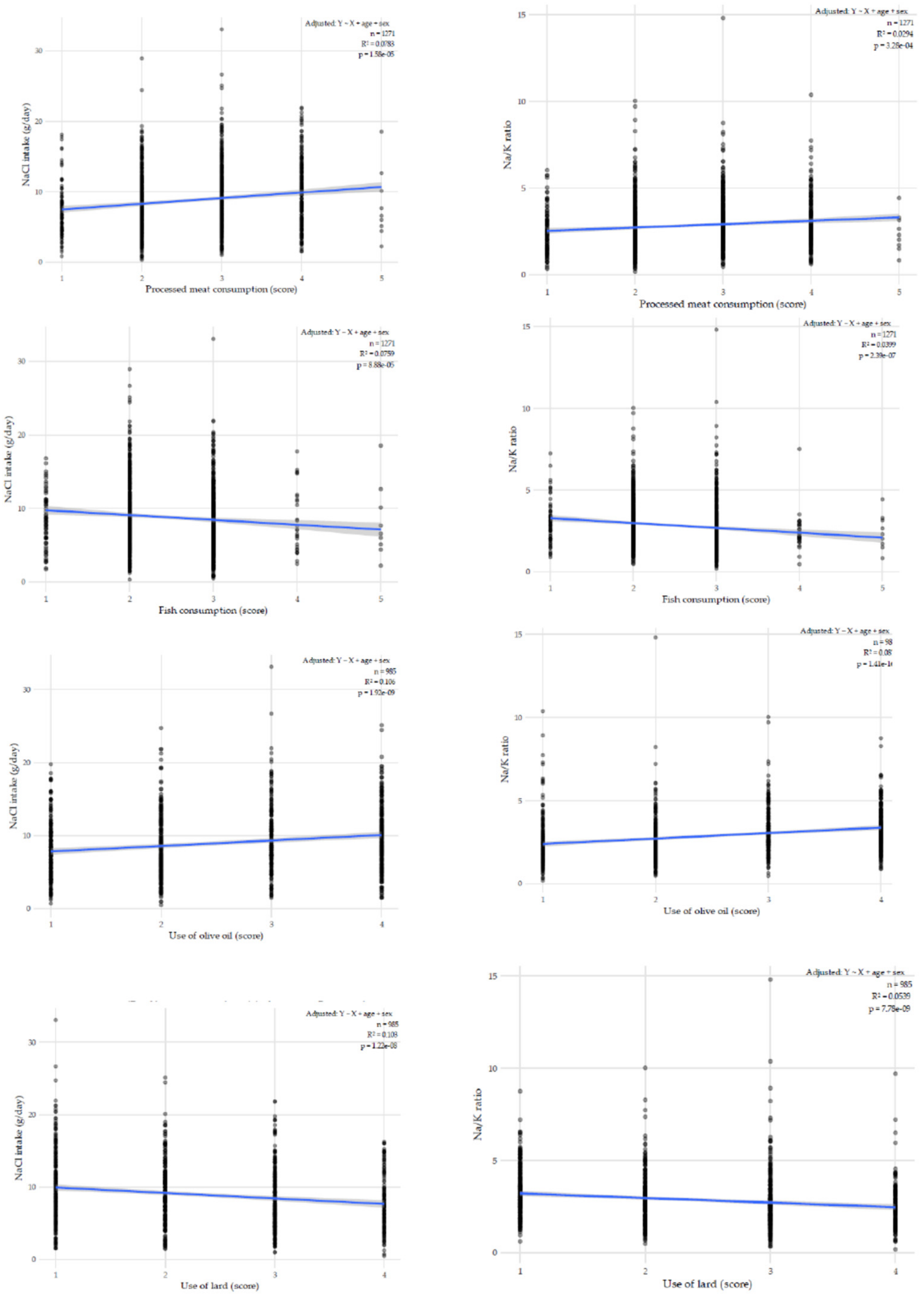

Figure S3. Spearman rank correlations between dietary habits and salt intake and sodium–to–potassium ratio, adjusted for age and gender.

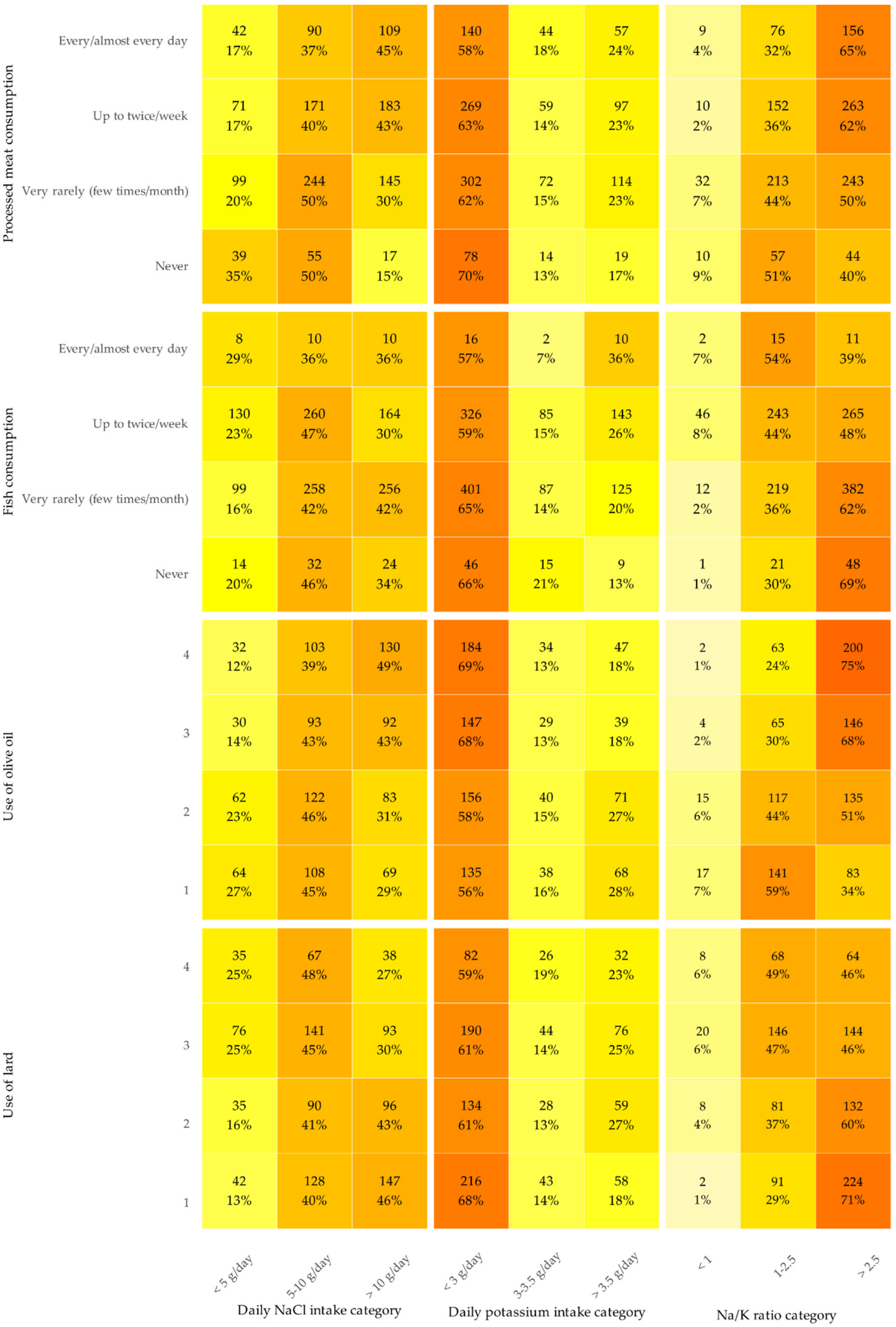

Figure S4. Heat maps of daily salt and potassium intake and sodium-to-potassium ratio categories according to the dietary habits.

Table S1. Relationship of risk factors with salt and potassium intake, and sodium-to-potassium ratio

	Higher potassium intake	Lower sodium-to-potassium ratio	Lower salt intake
Higher monthly income	0+	+	+
Higher personal qualification	0	+	+
Higher educational level	0+	0+	+
More physical activity	+	+	0
Smokers	-	-	+
Former smokers	+	-	-
Alcohol	0	0	0
Less processed meat	-	+	+
More fish	+	+	+
More olive oil	+	+	+

Green indicates healthy positive trend, red indicates healthy negative trend, yellow – no significant (neutral) trend
